# Genome-wide array comparative genomic hybridization analysis reveals distinct amplifications in osteosarcoma

**DOI:** 10.1186/1471-2407-4-45

**Published:** 2004-08-07

**Authors:** Tsz-Kwong Man, Xin-Yan Lu, Kim Jaeweon, Laszlo Perlaky, Charles P Harris, Shishir Shah, Marc Ladanyi, Richard Gorlick, Ching C Lau, Pulivarthi H Rao

**Affiliations:** 1 Texas Children's Cancer Center, Baylor College of Medicine, Houston, TX, USA; 2 Spectral Genomics, Houston, TX, USA; 3 Department of Pathology, Memorial Sloan Kettering Cancer Center, New York, New York, USA; 4 Department of Pediatrics, Memorial Sloan Kettering Cancer Center, New York, New York, USA

## Abstract

**Background:**

Osteosarcoma is a highly malignant bone neoplasm of children and young adults. It is characterized by extremely complex karyotypes and high frequency of chromosomal amplifications. Currently, only the histological response (degree of necrosis) to therapy represent gold standard for predicting the outcome in a patient with non-metastatic osteosarcoma at the time of definitive surgery. Patients with lower degree of necrosis have a higher risk of relapse and poor outcome even after chemotherapy and complete resection of the primary tumor. Therefore, a better understanding of the underlying molecular genetic events leading to tumor initiation and progression could result in the identification of potential diagnostic and therapeutic targets.

**Methods:**

We used a genome-wide screening method – array based comparative genomic hybridization (array-CGH) to identify DNA copy number changes in 48 patients with osteosarcoma. We applied fluorescence in situ hybridization (FISH) to validate some of amplified clones in this study.

**Results:**

Clones showing gains (79%) were more frequent than losses (66%). High-level amplifications and homozygous deletions constitute 28.6% and 3.8% of tumor genome respectively. High-level amplifications were present in 238 clones, of which about 37% of them showed recurrent amplification. Most frequently amplified clones were mapped to 1p36.32 (*PRDM16*), 6p21.1 (*CDC5L*, *HSPCB*, *NFKBIE)*, 8q24, 12q14.3 (*IFNG*), 16p13 (*MGRN1*), and 17p11.2 (*PMP22 MYCD, SOX1*,*ELAC27*). We validated some of the amplified clones by FISH from 6p12-p21, 8q23-q24, and 17p11.2 amplicons. Homozygous deletions were noted for 32 clones and only 7 clones showed in more than one case. These 7 clones were mapped to 1q25.1 (4 cases), 3p14.1 (4 cases), 13q12.2 (2 cases), 4p15.1 (2 cases), 6q12 (2 cases), 6q12 (2 cases) and 6q16.3 (2 cases).

**Conclusions:**

This study clearly demonstrates the utility of array CGH in defining high-resolution DNA copy number changes and refining amplifications. The resolution of array CGH technology combined with human genome database suggested the possible target genes present in the gained or lost clones.

## Background

Osteosarcoma (OS) is a primary malignant tumor of bone arising from primitive bone-forming mesenchymal cells and it accounts for approximately 60% of malignant bone tumors in the first two decades of life [1]. These tumors typically arise in the metaphyseal regions of long bones, with the distal femur, proximal tibia and proximal humerus. A significant number of osteosarcomas are of conventional type which can be subdivided into three major categories based on their predominant differentiation of tumor cells: osteoblastic, chondroblastic, and fibroblastic. Currently, only the histological response (degree of necrosis) to therapy represent gold standard for predicting the outcome in a patient with non-metastatic osteosarcoma at the time of definitive surgery [2]. Patients with lower degree of necrosis have a higher risk of relapse and poor outcome even after chemotherapy and complete resection of the primary tumor. Therefore, a better understanding of the underlying molecular genetic events leading to tumor initiation and progression could result in the identification of potential diagnostic and therapeutic targets.

Chromosomal aberrations in osteosarcoma are highly complex and characterized by high frequency of amplifications. These amplifications may result in the overexpression of genes and contribute to the genomic instability in osteosarcoma. The identification of genes within the amplified sites is crucial for understanding the biology and clinical behavior of osteosarcoma. Until, recently gene amplification has been detected by PCR, southern blot analysis or FISH-based approach using gene specific probes. These techniques are inherently restricted to the previously known amplified genes in the genome. In contrast, genome-wide screening of amplified chromosomal regions with CGH has become an important tool for the detection of amplified regions in the tumor genome. So far published chromosomal CGH studies in osteosarcoma have identified several high-level chromosomal amplifications at 1p22, 1p31, 1q21, 1q23, 2q24, 3p25, 3q26, 6q24.3, 4q12, 5p14-p15, 5q33, 6p12-p21, 6q24.3, 7p21-p22, 8q12-q23, 10p21, 10q11.1, 10q22, 11q13, 11q23, 12p13, 12q12-q15, 17p11.2, 17q21, 18q22, 19p13.1 and 20p11.2 [3-7]. However, conventional CGH has limited sensitivity and resolution (~10–15 megabases) because of its dependence on the morphology of metaphase chromosomes. In addition, extensive follow-up work is required to identify candidate genes after regions of gain or loss have been identified. Recently, novel method termed as array-based comparative genomic hybridization (array CGH) has been described, which enables high throughput quantitative measurement of high-resolution DNA copy number changes throughout the genome [8]. This method is based on hybridization of differentially labeled test and reference DNAs to an array of mapped human genomic DNA fragments (~100–200 kb) and has been recently applied to human and mouse tumors [9-14]. To identify high-resolution copy number, we used array CGH to the panel of 48 tumors. The resolution of array CGH technology combined with human genome database not only allowed a precise identification of amplicons but also suggested the possible target genes within the amplicons.

## Methods

### Patient samples

A total of 48 tumors from 42 patients (20 males and 22 females) were collected from the Texas Children's Cancer Center, Houston, TX (tumors 193, 196, 204, 209, 221, 226, 248, 274, 295, 311, 326, 341, 345, 360, 400, 464, 481, 501, 527, 591 and 606) and Memorial Sloan Kettering Cancer Center, New York (tumors 06, 15, 24, 25, 27, 29, 32, 34, 40, 48, 68, 76, 78, 79, 80, 82, 83, 85, 88, 95, 98, 99, 102, 123, 423, 425, and 474). All tissues in this study were obtained after IRB approved informed consents were signed. The age at diagnosis ranged from 5 years to 71 years at diagnosis. The histological information of 42 patients is presented in Table 1.

### Array CGH

The array used in this study consists of 967 human BACs, which were spaced approximately 3 megabase across the whole genome. These arrays were obtained from Spectral Genomics, Houston, TX. The experiments were performed according to the manufacturer's protocol. Arrays were pre-hybridized with human Cot-I DNA (GIBCO Invitrogen, Carlsbad, CA) and salmon testes DNA to block the repetitive sequences on BACs. One microgram of normal DNA (reference) and tumor DNA (test) was labeled with cy5-dUTP and cy3-dUTP respectively, by random priming. To avoid dye bias, we performed dye swap experiments for each sample. The probe mixture is dissolved in hybridization mixture, denatured, cooled, and mounted with 22 × 60 mm coverslip. Hybridizations were performed in sealed chambers for 16–20 hours at 60°C. After post hybridization washes, arrays were rinsed, dried with compressed air, and scanned into two 16-bit TIFF image files using Gene Pix 4000A two-color fluorescent scanner (Axon Instruments, Inc., Union City, CA) and quantitated using GenePix software (Axon Instruments, Union City, CA).

### Data processing and analysis

After scanning of the slide, the fluorescent intensities of the green and red channels were background subtracted. The resulting values were normalized by intensity based local weighted regression method (Lowess) to correct for systematic bias in dye labeling and fluorescent intensity [15]. Then the ratio of the red/green channel of each clone was calculated and log base 2 transformed (log ratios). Each experiment was repeated once with dye reversal to addressing the confounding effect of the dye and experiment. The average of the dye-reversal experiment pair was calculated by reversing the sign of one experiment so that the log ratio reflects the tumor versus normal ratio.

We developed a new analytical method called invariant analysis to define the significant copy number changes. This method is designed to: i) increase the power of the analysis by combining all the cases in our dataset to define an invariant population (unchanged population); and, ii) to address the signal to noise differences among individual cases due to sample and hybridization variability. Our goal is to define a set of unchanged clones that can be used to calculate the upper and lower bound thresholds of the log ratios for the unchanged population in each experiment. First, we calculated the variance of each clone from all the experiments. We computed the *p-values *of the each clone by comparing to the clone with median variance using chi-square distribution . The clones that have p-value greater than preset cutoff 0.9 were considered as invariant clone set, i.e. clones that do not vary significantly in all experiments. Then the mean and standard deviation of the log ratios of these invariant clones in each experiment were calculated. The clones with log ratios that exceed mean +/- 2 × SD of the invariant set were considered gains and losses, respectively. For amplification and homozygous deletions, clones were defined to have at least 2 fold of the upper bound threshold and 4-fold of lower bound threshold, respectively. The gene(s) present in the clones were identified using UCSC browser  by downloading gene table (refFlat) from human gene assembly, July 2003. We search the candidate genes based on linear mapping position, which include 100 kb up and downstream from the clone center position. The supplemental data for this article is available at: 

### Statistical analysis

Significant clones in 6p, 8q, 12q and 17p amplicons were calculated using 2-sample t-test with randomized variance model . The experiments in each of the two groups, amplification and normal, used for comparison were defined based on the invariant analysis (see above). The clones that have *p <0.001 *were considered as significant. We chose a stringent cutoff to minimize the multiple testing problem.

### FISH

FISH was performed to validate and quantify chromosomal amplicons using clones from 6p12-p21 (RP11-91E11, AL391415, RP11-81F7, RP11-79I2, RP11-90H17 and RP11-79F13), 8q24.3 (RP11-89K10), and 17p11.2-p12 (RP11-64B12, RP11-89K6 and RP11-189D22 on tumors metaphase/interphase cells from cases 274, 364, 425, 426, 527 and 628. We confirmed the map positions of all clones on normal human metaphase cells by FISH. The bacterial artificial chromosome (BAC) clones, and centromeric clone from 6 (pEDZ6) were labeled with Spectrum Red or Spectrum Green (Vysis, Downers Grove, IL) by nick translation. Hybridization and FISH analysis was performed as described previously [16].

## Results

To define the gains and losses in our experiments, we used invariant analysis for the first time to describe genomic changes by array CGH. In this method, we defined an invariant clone set that has low variance of log ratios among all the array experiments. After the mean and standard deviation of the log ratios in the invariant set of each experiment were calculated, clones that have higher or lower log ratios than the mean +/- 2SD of the invariant set (upper bound and lower bound) were used to define gains and losses. We chose to use this method because it addresses some of the shortcomings of the modeling method, such as using all information provided in a set of experiment to determine the unchanged population instead of using one experiment at a time. However, the variation of each experiment is accounted for because the thresholds are calculated using the invariant set from each experiment. It also does not require a separate reference set for comparison. Finally, it provides an adjustable cutoff to optimize the thresholds to the training data, if provided.

**Figure 1:**
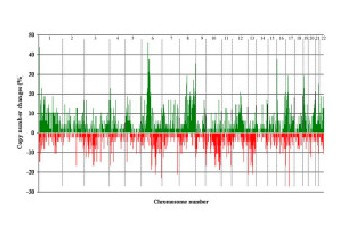
Frequency of DNA copy number changes identified by array CGH in 48 osteosarcomas. The gains and losses are depicted as green and red color bars respectively. Clones are arranged from chromosome 1 to 22 and within each chromosome on the basis of UCSF mapping position.

The amplified and homozygously deleted clones were defined to have at least 2 fold of the upper bound and 4-fold of lower bound, respectively. Figure 1 summarizes the high-resolution DNA copy number changes identified by array CGH in 48 osteosarcomas derived from 42 patients. Copy number changes were detected involving small genomic regions, whole chromosomes, and chromosomal arms showing homozygous deletions and high-level amplifications.

### Overview of genomic profiles

Copy number changes excluding clones from sex chromosomes were involved in a significant fraction of most tumor genome. The estimated average genomic distance between clones was ~3–4 Mb. The frequency of clones showing gains (79%) was greater than losses (66%). High-level amplifications and homozygous deletions constitute 28.6% and 3.8% of tumor genome respectively. The most frequently deleted clones were identified from the chromosomal regions 2q31.1, 3p14.1, 4p16.2, 6q12, 6q21, 6q27, 7q35, 10p15.1, 10q22-q23, 10q25-q26, 11q25, 13q12.2, 13q14.3, 13q22.1, 17p13.3 and 17q12 (Table 2). Most frequently gained clones were mapped to chromosome 1p36, 4p16, 6p12-p21, 8q21, 8q23-q24, 12q14.3, 16p13.3, 16q24.3, 17p11-p12, 19p13.3 and 21q22.3 (Table 3). We explored the possible statistical relationship between copy number alterations and histological and clinical parameters. We found no significant relationship between copy number changes and primary/metastatic disease, or histological type or histological response. This may be due to the involvement of large number of genomic loci and insufficient sample size.

**Figure 2:**
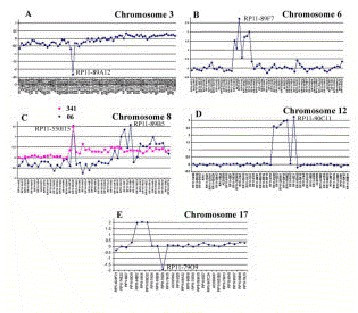
A representative chromosome profiles showing homozygous deletions (**A, E**) and high-level amplifications (**B- E**). The clones showing homozygous deletions from 3p14.1 (RP11-89A12) and 17q12 (RP11-79O9) in tumor 06 (**A, E**) and high-level amplifications from 6p12-p21 in tumor 248(**B**), 8q12 in tumors 06 and 341 and 8q23-q24 in tumor 06, (**C**), 12q13-q15 in tumor 48, (**D**) and 17p11.2 in tumor 06(**E**), are shown as log ratios (Y-axis). The clones on each chromosome are arranged (pter to qter) on the basis of UCSC mapping positions.

Homozygous deletions were noted for 32 clones (3.8%). Recurrent homozygous deletions were noted for 7 clones that are were mapped to 1q25.1 (4 cases), 3p14.1 (4 cases), 13q12.2 (2 cases), 4p15.1 (2 cases), 6q12 (2 cases), 6q12 (2 cases) and 6q16.3 (2 cases). Figure 2A is showing a homozygous deletion at 3p14.1 in tumor 06. Loss of 6q12 region was noted in 35% of the osteosarcomas. This region was covered with four clones spanning ~4.2 Mb. Two tumors (tumor 27 and 345) showed low intensity ratios indicting homozygous deletions in this region, one tumor (tumor 345) showed all 4 deleted clones spanning ~4.2 Mb with RP1-129L7 having the lowest ratio intensity decrease. In another case (tumor 27), two clones (RP1-46B1 and RP1-129L7) showed decreased intensity ratios indicating homozygous deletions. Both these clones spanning approximately 2.6 Mb of 6q12 region.

### Amplification is a frequent phenomenon in osteosarcoma

**Figure 3:**
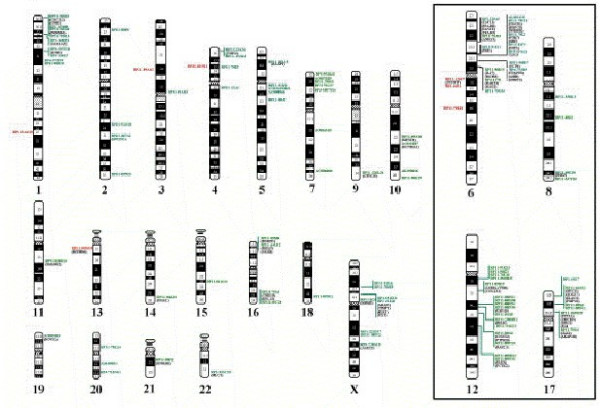
Ideogram showing recurrent homozygous deletions (left) and high-level amplifications (right) identified by array CGH in 48 cases. The gene(s) contained within the BAC clone are shown in parentheses of the respective clones. Gene(s) present in the BAC clones were identified using UCSC browser  by downloading gene table (refFlat) from human gene assembly, July 2003. The ideograms for chromosomes 6, 8, 12 and 17 are shown separately.

Previous studies using CGH have identified several chromosomal amplification sites in osteosarcoma. Because of the limitation of the method, it fails to pinpoint the precise site of amplicon. However, the present study by array CGH has identified 238 clones (28.6%) with high-level amplifications. Recurrent amplifications were noted in ~37% of the total amplified clones (Figure 3). These amplified clones were mapped to 1p22, 1p31.1 (*ROR1*), 1p36.1 (*PRDM16*), 1q21, 1q23 (*TNFF6*), 2q24, 3p25, 3q26.1, 4p16.3, 5p14, 5q33, 6p11.2-p21, 7p21, 8q12.1, 8q24.13, 10p21, 10q11.1, 10q22 (*KCNMA1*), 11q13, 11q23 (*GRIK4*), 12q12, 12q13-q15, 12q21-q21.33, 17p11.2-p12, 17q21 (*NGFR*), 18q22, and 19p13.1 (*NFAT). *Of these amplified sites, 6p11.2-p21, 8q12.1, 8q24.13, 12q12, 12q13-q15, 12q21-q21.33, 16p13 and 17p11.2-p12 were frequent.

**Figure 4:**
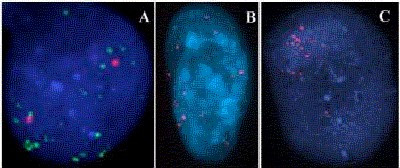
FISH validation of some of the high-level amplifications (6p12.1, 8q24.3 and 17p11.2) identified by array CGH. Interphase cells hybridized with centromere 6 (red)/RP11-81F7 (green) in case 274 **(A)**, RP11-89K10 (red) in case 527 **(B) **and RP11-189D22 (red) in case 364 **(C)**. The ploidy of these cases was determined based on the modal chromosome number of the respective cases, e.g. diploid (case 426) triploid (cases 274 and 364), and tetraploid (case 527).

Gain of clones from 6p12-p21 regions was noted in 33/48 (~65%) cases analyzed. High-level amplification of the clones from same region was noted in 25% of the cases by array CGH. We found that most of the cases with amplification of 6p12-p21 displayed either increased or slightly varying degree of copy number increase across the 6p12-p21 region. The combined log ratios from all the cases defined the boundaries of amplification between RP3-329A5 and RP11-79F13. The amplicon spans approximately 9.4 Mb with amplification peak for clone RP11-81F7. Further, we used FISH to validate 6p amplicon on tumor metaphase and interphase cells from cases 274, 364, 426 and 527. Increased copy numbers for clones RP11-91E11, AL391415, RP11-81F7, RP11-79I2, RP11-90H17 and RP11-79F13 were noted in interphase cells with maximum copy number increase for clone RP11-81F7 (Figure 4A). This was consistent with amplification peak for clone RP11-81F7 in the tumors profiled by array CGH (Figure 2B). In addition, we used 2-sample t-test with randomized variance model to define significant clones from 6p12-p21 amplicon. By this method, we identified RP11-79F13 (p = 0.00000007), RP11-79I2 (p = 0.00000007) and RP11-81F7 (p = 0.00000007) as statistically significant clones.

Most cases with 8q gain, displayed varying degree of copy number increase predominantly from 8q12.1 (16.9%), 8q21.13 (29%), and 8q24.3 (35%). High-level amplifications were also noted from 8q12.1 (RP11-550I15 – 6.3%; Figure 2C), 8q21.13 (RP11-89H1 – 6.3%), 8q24.3 (RP11-89K10 – 6.3%) and RP11-637F16 (12.5%). FISH using clone RP11-89K10 (p = 0.00049) on interphase cells from case 527 confirmed the amplification (10–12 copies) (Figure 4B).

Amplification of 12q was noted in 14/51 (~27%) tumors analyzed by array CGH. Three distinct amplicons – AMP1 (12q12), AMP2 (12q14.1) and AMP3 (12q21.33) were noted across the entire long arm of chromosome 12 (Figure 2D). Of these 14 cases, four of them (80, 123, 248, 341) displayed all three amplicons. The AMP1 was noted in 10 cases covering 1.8 Mb region between RP11-91K15 and RP11-90I21 with peak amplification for clone RP11-91K15 (p = 0.00000004). Another amplicon (AMP2) was noted 24.48 Mb distal to AMP1 between RP11-91K23 and RP11-89P15. The AMP3, which was 23.3 Mb distal to AMP2 containing RP11-89F6.

Amplification of 17p11.2 was noted in 27% of the cases analyzed by array CGH. The amplicon was composed of three clones RP11-64B12 (p = 0.0000014), RP11-89K6 (p = 0.00000005) and RP11-189D22 (p = 0.0000001) and covering 3.7 Mb region on the short arm of chromosome 17 (Figure 2E). We used these three clones as FISH probes to validate 17p amplicon in tumors 274, 364, 425 and 628 on interphase/ metaphase cells. The distribution of copy number for this amplicon in all the cases ranged from 4–14 copies with peak amplification for clone RP11-189D22 (10–14 copies), followed by and RP11-89K6 (8–10 copies) RP11-64B12 (6–8 copies) (Figure 4C).

## Discussion

This study represents the first application of genome-wide copy number changes by array CGH in osteosarcoma. Recent studies in breast, renal and bladder cancer showed the potential assessment of this technology in detecting high-resolution copy number changes [9,11,14]. This approach will augment the identification of cancer causing genes by relating the clone information directly with sequence information from human genome database. In this study, we used array CGH to screen for high-resolution DNA copy number changes and precise identification of amplifications in a panel of 48 osteosarcomas.

Gene amplification is an important genetic mechanism in human cancers, as it clearly associated with tumor progression and has a prognostic significance and has even provided a target for therapeutics [17,18]. These amplifications are often seen at the cytogenetic level as homozygously staining regions (hsrs) or double minute chromosomes (dms). However, cytogenetic recognition of amplifications doesn't contribute to the mapping and identification of amplified DNA sequences. The advent of CGH points an ever-increasing number of chromosomal amplifications in various tumors. These amplifications contribute to the genomic instability in tumors. We have recently shown that the mutation of *p53 *significantly correlates with genome-wide DNA instability and seems to represent a major genetic factor contributing to the extremely high levels of genomic instability found in high-grade osteosarcomas [19].

Our analysis have identified frequently amplified clones from 6p11.2-p21, 8q12.1, 8q24.13, 12q12, 12q13-q15, 12q21-q21.33, 16p13 and 17p11.2-p12. Amplification of clones from 6p12-p21 region was noted in 25% of the cases analyzed. This was consistent with the previously published results by CGH. By array CGH, we refined the 6p amplicon to 9.4 Mb with amplification peak for clone RP11-81F7. We recently demonstrated the origin of 6p amplicon as consequence of tandem duplication of clones RP11-81F7 and RP11-79F13 [7]. Based on combined array CGH and FISH analysis suggest *CDC5L*, *HSPCB*, and *NFKBIE*, and *HGNC *and *MRPL14 *are the target genes from 6p12-p21 amplicon. Of these genes, *CDC5L *may be an important gene in cancer because of its role as a positive cell cycle regulator for G2/M transition[20]. Consistent with our analysis, overexpression of *HSPCB *was shown recently by cDNA microarray studies on osteosarcoma [21]. This protein was shown to play an important role in assemble/disassembly of tubulin by inhibiting tubulin polymerization.

High-level amplifications were also noted from 8q12.1 (RP11-550I15 – 6.3%), 8q21.13 (RP11-89H1 – 6.3%), 8q24.3 (RP11-89K10 – 6.3%) and RP11-637F16 (12.5%). There were no candidate genes present in clones RP11-550I15, RP11-89H1 and RP11-637F16, but clone RP11-89K10 contained *NSE2 *(breast cancer membrane protein 101 kDa) gene.

High-level amplification of clones on 12q revealed three distinct sites of amplifications – AMP1 (12q12), AMP2 (12q14.1) and AMP3 (12q21.33). Pervious studies have shown the amplification *GLI, CHOP, SAS, HMGI-C, CDK4*, *HDM2, *and *PRIM1 *from 12q13-q15 region in osteosarcoma [22,23]. The present array CGH analysis identified a possible target gene *IFNG *from AMP2 (RP11-298M11; p = 0.0000001), which is physically mapped close to the *HDM2 *oncogene locus[24]. Previous studies demonstrated that T-cell production of *IFNG *strongly suppresses osteoclastogenesis by interfering with the RANKL-RANK signaling pathway. *IFNG *induces rapid degradation of the RANK adaptor protein, TRAF6, resulting in strong inhibition of the RANKL-induced activation of the transcription factor NFKB and JNK [25]. The AMP3, which was 23.3 Mb distal to AMP2 containing RP11-89F6. Our analysis from AMP3 revealed two interesting candidate genes: transcription factor *ELK3 *and PCTAIRE protein kinase 2 (*PCTK2*). *ELK3 *is a member of the ETS-domain transcription factor family and the protein is activated by signal-induced phosphorylation [26]. The protein encoded by *PCTK2 *belongs to the cdc2/cdkx subfamily of the ser/thr family of protein kinases and play an important role in the regulation of the mammalian cell cycle [27]. High-level amplification of three clones from 12p13 was noted in case 27 and the amplicon span 4.6 Mb with peak amplification for clone RP11-89D16. No candidate genes contained with in this BAC. Amplification 12p has been reported previously in 9/19 high-grade osteosarcomas by CGH. Recent FISH analysis has identified the amplification of *CCND2, ETV6*, and *KRAS2 *from 12p region [28].

Amplification of 17p11.2 was noted in 27% of the cases analyzed by array CGH. Our array CGH analysis has identified three clones with high-level amplifications that spans ~3.7 Mb region on 17p11.2. Several candidate genes were identified within these clones (T*PP3A, SMCR5, DRG2, FL11*, *MYCD, SOX 17, ELAC2*, and *PMP22*). Recent studies have shown the amplification of some of the genes identified in the present study (*PMP22, *and *TOP3A*) from 17p11.2-p12 in high-grade OS by semi-quantitative PCR and cDNA microarrays [29,30].

The present array CGH analysis has identified seven recurrent clones exhibiting homozygous deletions from 1q25.1, 3p14.1, 13q12.2, 4p15.1, 6q12, 6q12 and 6q16.3. These chromosomal regions were consistent with previously reported studies by loss of heterozygosity (LOH) and CGH [3-7,31]. The clone, RP11-90M15 (13q12.2) contain possible candidate gene *MTMR6*, a protein-tyrosine phosphatase gene and shown to be present within a cloned region that encompasses a translocation breakpoint t(8;13) in an atypical myoproliferative disorder [32]. Homozygous deletions of two clones spanning approximately 2.6 Mb of 6q12 region containing candidate genes – nuclear fragile X mental retardation protein interacting protein 1 pseudogene (*NUFIP1P*) and *BAI3 *gene (brain-specific angiogenesis inhibitor gene), which is to homologous to *BAI1 *and shown to suppress glioblastoma [33].

## Conclusions

In summary, high resolution array-based CGH revealed large number of chromosomal aberrations previously identified in osteosarcoma by chromosomal CGH and conventional cytogenetic methods. The present study allowed precise identification of smaller DNA copy number alterations, which suggest the presence of specific target genes in osteosarcoma. Although this study suggested several possible target genes from amplified regions from 6p, 8q, 12q and 17p, but these genes should be validated by other molecular and immunohistochemical approaches on well-defined large patient samples. Further, interaction or association studies between small genomic losses and gains will facilitate the identification of new genetic pathways in the pathogenesis of osteosarcoma.

## Competing interests

None declared.

## Authors contributions

TKM and KJ have contributed towards the data analysis. LP, ML, RG, and CL were assisted in sample collection and clinical information of the patients. X-YL has involved in array CGH experiments and data collection. CPH has involved in extracting the gene information from BAC clones. SS has provided the arrays used in this study. PHR was involved in the planning, and organization of the project.

## Pre-publication history

The pre-publication history for this paper can be accessed here:



## Figures and Tables

**Table 1:** Histological information on 48 osteosarcoma samples

Tumor No.	Sex	Age-Dx	Site	Histological Subtype	Type	Metastatic Disease	Huvos Grade	Response
06	Female	7	Distal Femur	NA	Biopsy	L	II	PR
15	Male	35	Distal Femur	Osteoblastic+MFH-Like	Biopsy	L		
24	Female	16	Clavicle	Chondroblastic	Biopsy	L	Minimal	
25	Male	8	Skull	Osteoblastic	Mets	L		
27	Female	34	Ischium	Mixed Chondroblastic+Fibroblastic-Like	Mets	L		
29	Male	19	Ilium	NA	Biopsy	M	II	PR
32	Female	25	Ilium	Chondroblastic	Biopsy	L	Minimal	
34	15	Femur	Chndroblastic And Osteoblastic	Biopsy	L	II	PR	
40	Female	11	Left Distal Femur	Giant Cell	Definitive	None	IV	GR
48	Female	32	Proximal Tibia	Osteo/Fibro/Chondroblastic	Definitive	None	I	PR
68	Male	18	Femur	Telangectactic	Mets			
76	Female	20	Thigh	N/A	Biopsy	L		
78	Male	5	Humerus	N/A	Biopsy	L	III	GR
79	Male	9	Tibia	Telangectactic	Mets	L	III	GR
80	Male	46	Tibia	N/A	Biopsy	L	I	PR
82	Male	23	Humerus	Osteoblastic	Mets	L	I	PR
83	Female	12	Femur	Telangiectatic	Biopsy	L	IV	GR
85	Male	34	Femur	Fibroblastic	Biopsy	L	I	PR
88	Male	17	Humerus	Chndorblastic	Biopsy	L	IV	GR
95	Male	71	Femur	Giant Cel Rich	Biopsy	L		
98	Female	31	Ilium	Epithelioid	Biopsy	L		
99	Female	22	Humerus	N/A	Biopsy	L		
102	Male	70	Humerus	Fibrohistiocytic	Mets	L		
123	Female	16	Femur	N/A	Biopsy	L	I	PR
209	Female	17	Distal Femur	Osteoblastic	Biopsy	No	II	PR
221	Female	17	Femur	Osteoblastic	Biopsy	No	IV	GR
248	Female	13	Tibia	Pleiomorphic	Biopsy	No	?	
311	Female	13	Distal Femur	Osteoblastic	Definitive	No	III	GR
326	Female	20	Femur	Osteoblastic	Pul Met	Yes	IV	GR
341	Male	12	Lemur	Fibroblastic	Biopsy	N	II	PR
345	Male	10	Distal Femur	Osteosarcoma	Biopsy	No	IV	GR
360	Female	18	Distal Femur	Osteoblastic	Biopsy	Yes	IV	GR
400	Female	15	Distal Femur	Chondroblastic	Yes			
423	Male	30	Proximal Humerus	Giant Cell	Definitive	None	I	PR
425	Male	24	Tibia	N/A	Biopsy	Proximal Femur	I	PR
474	Female	17	Pelvis	Chondroblastic	Biopsy	None	II	PR
591	Male	15	Proximal L Tibia	Telangectactic	Definitive	No	II	PR
193a	Female	17	Distal Femur	Osteoblastic	Biopsy	No		
196a	Female	15	Distal Femur	Osteoblastic	Pul Met	Yes		
274b	Male	13	Distal Femur	Osteoblastic//Chondroblastic	Biopsy	No	II	PR
295b	Male	13	Femur	Osteoblastic//Chondroblastic	Yes	II	PR	
464c	Female	15	Distal Femoral Head	Osteoblastic/Spindle Cell/Chondroblastic	Biopsy	No	II	PR
501c	Female	15	Femur	Osteoblastic	Definitive	No	II	PR
606c	Female	14	Distal Femur	Osteoblastic	Biopsy	Yes	II	PR
481d	Male	10	Distal Femur	Osteoblastic	Biopsy	No	II	PR
527d	Male	10	Proximal Tibia	Osteoblastic	Definitive	Yes	III	GR
204e	Female	18	Distal Femur	Osteoblastic	Biopsy	No	I	PR
226e	Female	18	Distal Femur	Telangectactic	Definitive	No	I	PR

^a, b, c, d, e-^Tumor samples obtained from the same patient.

**Table 2:** Most frequently lost clones.

Clone	**Map **Position (Mb)	Cyto Position	Frequency (%)	Genes
RP11-79K15	31.9	17q12	27	
RP1-140C12	170.4	6q27	22.9	*PSMB1*
RP11-90M15	24.8	13q12.2	20.8	
RP11-79I4	73.4	13q22.1	20.8	*KLF12*
RP11-79K22	101.7	6q16.3	20.8	*MTMR6, NUPL1*
AC004889	148.401	7q35	20.8	*OR2A4*
RP11-89H7	116.5	10q25.3	18.8	
AL359836	128.22	10q26.11	18.8	
RP11-80L16	67	6q12	18.8	
RP11-80D10	2.8	10p15.1	16.7	
AC021027	84.029	10q22.3	16.7	
RP11-79E24	88	10q23.2	16.7	
RP11-90B19	131.5	10q26.3	16.7	
RP11-835G21	143.17	11q25	16.7	
RP11-80H2	50	13q14.3	16.7	
RP11-81D9	72.8	13q22.1	16.7	
RP5-1029F21	1.21	17p13.3	16.7	
AC020681	175.95	2q31.1	16.7	*PDK1*
RP11-89A12	68	3p14.1	16.7	
RP11-9A1	71.28	3p14.1	16.7	
RP11-492I23	3.64	4p16.2	16.7	
RP1-46B1	69.4	6q12	16.7	*BAI3*
RP3-454N4	106	6q21	16.7	

**Table 3:** Most frequently gained clones.

Clone	Map Position (Mb)	Cyto Position	Frequency (%)	Genes
RP11-81F7	43.8	6p21.1	45	*HGNC, MRLP14*
RP1-163G9	2.6	1p36.32	43	*PRDM16*
RP11-79F13	44.6	6p21.1	37.5	*CDC5L, HSPCB, NFKBIE*
RP11-90H17	46.5	6p12.3	37.5	*UCP4*
RP11-64L12	0.68	16p13.3	37.5	*MSLN, SOX8*
RP11-637F16	144.61	8q24.3	35.4	
RP3-447E21	46	6p21.1	35.4	*CLIC5*
RP11-79I2	43.4	6p21.1	31.2	*EGFL*
RP11-80F24	78.3	8q21.13	29	
RP4-753D5	50.9	6p12.3	29	*TFAP2B*
AC005263	0.95	19p13.3	29	*AMH, GNRPX, DIT1L*
RP11-189D22	19.56	17p11.2	29	*TPP3A, SMCR5, DRG2, FLI*
RP11-89P19	119.5	8q23.3	27	
RP11-89H1	77.4	8q21.13	25	
RP11-88N2	43.7	21q22.3	25	*SNF1LK*
RP1-283E3	1.5	1p36.33	25	*CDC2L2, GNB1*
RP11-89P9	125.4	8q24.13	22.9	*MTSS1*
AL391415	43.06	6p21.2	22.9	*GLO1, DNAH8*
RP11-492I23	3.64	4p16.2	22.9	
RP1-163M9	16.2	1p36.13	22.9	
RP11-383B15	2.89	19p13.3	22.9	
RP11-89K6	13.1	17p12	22.9	
RP11-89K10	127.3	8q24.13	20.8	*NSE2*
RP11-90D11	98.8	8q22.1	20.8	
RP11-91E11	37.4	6p21.2	20.8	*PIM1*
RP3-417I1	63.04	6p11.2	20.8	*BAG2, RAB23*
RP11-79O4	19.9	17p11.2	20.8	*ULK2, AKAP10*
RP11-46C24	101.27	16q24.3	20.8	
RP11-91K23	67	12q14.3	20.8	
